# The Registration of Nursing Homes

**Published:** 1907-11-16

**Authors:** 


### THE REGISTRATION OF NURSING HOMES.

### A NATIONAL QUESTION.

"In the event of even the mildest measure for the regulation of nursing homes becoming law, 90 per cent. of these establishments would be compelled to close their doors." This is the dictum of one who has had the widest opportunities for forming a judgment on this matter, and we doubt if the most optimistic observer would be prepared to dispute the assertion. All that the best architects can devise, all that scientific investigations have proved to be most conducive to recovery, are lavished on the buildings in which the poor are nursed back to health. Operations are performed in surroundings from which the invading microbe has been excluded by a ceremonial far exceeding in strict observance of ritual the most exacting form of religion. Every article composing the bed in which the patient is placed has been scrutinised, purified, specially prepared to serve its purpose. The nurses are the most highly skilled the country affords, the diet is carefully regulated, the air is warmed, and religiously guarded from any possible source of septic infection. The consequences are apparent. To operate is to succeed. Recovery tends to become more and more rapid, and the hospital beds are emptied in quick succession of convalescents in order to receive fresh sufferers.

How are the *supporters* of the hospitals nursed in their own illness? Their operations, performed by the same surgeons, take place in any room which happens to be vacant in the nursing home to which they have been consigned. It may have been tenanted by a highly septic case only a day or two before. It may merely have been used as a sleeping apartment for an extra nurse on an emergency. It is hastily brushed down, and the patient must take his chance, for it is the best that offers. What becomes of the *débris* which results when the operation is concluded? In the hospital a trained sister, with a probationer and special wardmaid, are kept busy removing the traces of the preceding operation and cleansing the theatre before the next operation takes place. In the nursing home a hurried nurse reduces the apartment to order, perhaps for the reception of some urgent case just coming in, and the details resulting from the operation are collected and set aside in some secret receptacle until late at night, when, servants and nurses being safe in bed, the proprietress can steal downstairs and empty the contents on to the kitchen fire, where all the meals for the household are to be cooked on the morrow. Meantime the patient is removed to the apartment which the means at his disposal have enabled him to hire for the time being. If he can afford to pay from eight to fifteen guineas a week, probably the air-space may approximate to what the patient gets in hospital. But who has inquired into the character of the illness last nursed in that room? What system of disinfection has taken place since that recent death from cancer in that very bed? Has the bedding been changed? Have even the blankets been washed? In what condition are the drains? All this has been left to the lady at the head of the establishment, and the easy belief is entertained that, being herself a trained nurse, she is capable of fulfilling the duties which, in hospital, task the energies of medical superintendent, house surgeon, secretary, and matron. Probably she has been very busy all day receiving and discharging patients, giving orders to the servants, interviewing distracted relatives, and explaining to medical men the perfections of her home. She has given orders to her one trained assistant to prepare the room for a new patient; and if the "sister" has done only what seemed strictly necessary to an overworked mind, the fault can hardly be laid on her. A special nurse is placed in charge of the critical case. She may be "special," but it is in the manner of her training in that very establishment. Thus attended, sharing during the night the attentions of the half-trained night nurse who serves the whole house, the patient worries through. Perhaps he slowly and uncomfortably recovers, and comes out to swell the loud public complaints against "hospital nurses" and nursing homes. Perhaps he does not recover, but in that case no one seems at all surprised. He has "sunk under the operation," or "failed to rally." His bed is empty, and the very next day it is filled by someone else, fatally disqualified by the possession of an independent income from receiving proper treatment.

Nothing less than a strong wave of popular feeling can serve to end the abuses which have been thus barely indicated. If the public could only be brought to realise the tale of misery inflicted by all the long-retarded recoveries, by all the preventable deaths, directly due to mismanaged nursing homes, they would call unanimously for a measure which should remedy an abuse daily growing to more alarming proportions. Had these helpless victims been destitute their cause had long since been pleaded effectually. May we not dare for once to plead for the rich, the wise, and the benevolent? Will not the chairman of one of our great voluntary hospitals gather round him a strong committee charged with the duty of drawing up suitable regulations for the conduct of nursing homes, with a view to the introduction of a Bill for their registration? Not a voice would be raised against the proposal, for the really well-managed nursing homes—of which there are a few—are themselves eager for registration, and the others would not dare to proclaim the fact of their own deficiencies. Surely it is time that the generous supporters of voluntary hospital work should share in the advances of science, and reap benefits they have conferred with open hands on the poor. The world is progressing far on the road of altruism when the poor are in safety while the rich are neglected. Were it not well to remember the precept of the good Physician: "This ought ye to have done, and not to leave the other undone"?

